# A Promiscuous Bacterial P450: The Unparalleled Diversity of BM3 in Pharmaceutical Metabolism

**DOI:** 10.3390/ijms222111380

**Published:** 2021-10-21

**Authors:** Sian Thistlethwaite, Laura N. Jeffreys, Hazel M. Girvan, Kirsty J. McLean, Andrew W. Munro

**Affiliations:** 1 Manchester Institute of Biotechnology, University of Manchester, 131 Princess Street, Manchester M1 7DN, UK; sian.thistlethwaite@manchester.ac.uk; 2 Centre for Drugs and Diagnostics, Department of Tropical Disease Biology, Liverpool School of Tropical Medicine, Liverpool L3 5QA, UK; Laura.Jeffreys@lstmed.ac.uk; 3 Department of Biological and Geographical Sciences, School of Applies Sciences, University of Huddersfield, Queensgate, Huddersfield HD1 3DH, UK; H.M.Girvan@hud.ac.uk (H.M.G.); K.J.Mclean@hud.ac.uk (K.J.M.)

**Keywords:** CYP102A1, P450 BM3, drug metabolism, cytochromes P450, P450 engineering, biocatalysis

## Abstract

CYP102A1 (BM3) is a catalytically self-sufficient flavocytochrome fusion protein isolated from *Bacillus megaterium*, which displays similar metabolic capabilities to many drug-metabolizing human P450 isoforms. BM3′s high catalytic efficiency, ease of production and malleable active site makes the enzyme a desirable tool in the production of small molecule metabolites, especially for compounds that exhibit drug-like chemical properties. The engineering of select key residues within the BM3 active site vastly expands the catalytic repertoire, generating variants which can perform a range of modifications. This provides an attractive alternative route to the production of valuable compounds that are often laborious to synthesize via traditional organic means. Extensive studies have been conducted with the aim of engineering BM3 to expand metabolite production towards a comprehensive range of drug-like compounds, with many key examples found both in the literature and in the wider industrial bioproduction setting of desirable oxy-metabolite production by both wild-type BM3 and related variants. This review covers the past and current research on the engineering of BM3 to produce drug metabolites and highlights its crucial role in the future of biosynthetic pharmaceutical production.

## 1. Context and Introduction to BM3

Most commercially available pharmaceuticals are produced via traditional organic synthesis routes. However, this approach is limited by the scope of traditional chemical catalysts that often yield predictably similar products. This leads to a difficulty in the synthesis of compounds that exhibit enantioselectivity and structural motifs such as specific hydroxyl groups. There is also a commercial interest in reducing manufacturing costs. Due to these factors, efforts are being made to move future pharmaceutical synthesis towards alternative routes of production that focus on sustainability and cost efficiency [1]. One area of current interest is biosynthetic manufacture, which harnesses bacterial cells and enzymes for metabolite production as an organic synthesis replacement. These alternatives are both relatively cheap and easy to produce. Additionally, enzymes often have the added advantage of exhibiting higher yields and selectivity towards native small molecule substrates. Of particular interest are the cytochrome P450 (P450 or CYP) enzyme superfamily. These heme enzymes are primarily monooxygenases that are found in most living organisms and share similarities in their characteristic ‘P450′ fold and the highly conserved, cysteine thiolate-coordinated heme prosthetic group [2].

P450s perform oxidations on a diverse range of organic small molecule substrates and have many physiological roles in biosynthetic and detoxification pathways [3]. A major biotransformation that is typical of the P450 superfamily is the hydroxylation of carbon–hydrogen bonds via the insertion of an oxygen atom, yielding a hydroxylated metabolite and a molecule of water. The metabolism of substrates is achieved by a complex multi-step catalytic cycle via the heme prosthetic group. In this mechanism, the docking of the substrate and the removal of the distal water ligand of the heme iron triggers a series of transient changes in the heme iron state. These changes involve the transfer of two electrons, a proton and the binding of dioxygen. In the canonical catalytic cycle this leads to the eventual abstraction of a substrate proton which then rebounds to produce an oxidized metabolite [4]. Other P450-catalyzed biotransformations include (but are not limited to) heteroatom oxygenations, dealkylations, epoxidations, ring cleavages, dehydrogenations and sulfoxidations [3]. Human P450s have numerous physiological functions and are widely known for their role in xenobiotic metabolism, typically adding polar functional groups, facilitating an excretion of the product [5]. Since the discovery of P450s there has been an interest in utilizing the P450 superfamily as an alternative route for the synthesis of valuable small molecules, as their mechanistic capabilities can yield oxy-metabolites that are laborious to synthesize via traditional organic synthesis routes. The main drug-metabolizing human P450s exhibits promiscuity and wide substrate profiles. However, due to their eukaryotic nature and N-terminal membrane anchor they can be difficult to express in bacterial hosts that are typically used for biocatalytic cascades. Furthermore, their functioning requires the use of a reductase partner to supply the electron necessary for catalysis. In general, human P450s are catalytically slow. Therefore, there is an advantage to be gained from utilizing soluble bacterial P450s as biocatalysts for metabolite production.

### P450 BM3

A bacterial P450 that has been explored extensively for metabolite production capabilities is the natural fusion protein, flavocytochrome P450 BM3 (BM3, CYP102A1) [6,7,8]. BM3 was isolated in the early 1970s from the soil bacterium, *Bacillus megaterium* (the third P450 to be extracted from *B. megaterium*), and was identified as a fatty acid hydroxylase that did not require the addition of an independent redox partner [6,7]. Structural studies reveal that BM3 is a 119.5 kilodalton (kDa) single polypeptide enzyme of 1048 amino acids that forms a soluble dimer in solution. It contains a 55 kDa heme domain that is fused via a short linker region to a 65 kDa reductase domain that houses FAD and FMN flavins [9,10]. An interesting observation is that both domains exhibit structural similarities to eukaryotic microsomal P450-containing systems. Specifically, there is the similarity of the reductase domain to human cytochrome P450 reductuase (CPR) and of the heme domain to the CYP2A and CYP4A fatty acid hydroxylase sub-families, while there is a lack of the N-terminal encoded transmembrane anchor [11,12]. P450 BM3′s precise function in vivo is not particularly well defined, but it is postulated that it plays a role in the detoxification of xenobiotic lipids produced by plants [13,14].

**Figure 1:**
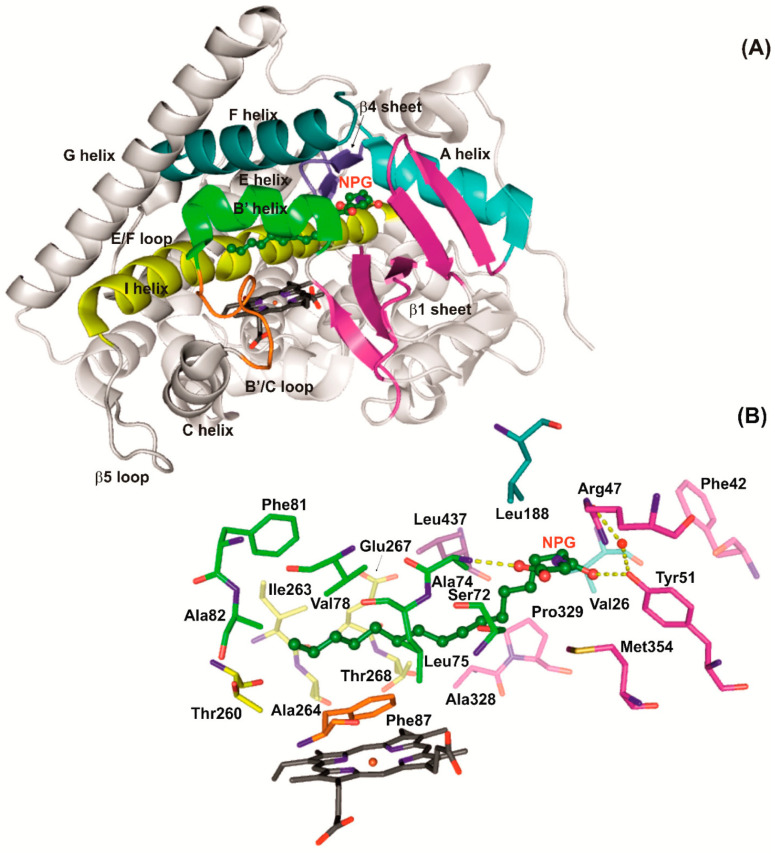
The secondary structure and active site of NPG-bound WT BM3 heme domain (PDB: code 1JPZ). (**A**) Secondary structure of WT BM3 with key regions indicated. The heme group is shown as dark grey sticks with the iron in brown and NPG shown as ball and sticks in dark green. Regions that form the active site and substrate access channel are in multi-colors. (**B**) The substrate access channel and active site of NPG-bound WT BM3. Heme and NPG are indicated as in panel A. All oxygens are in dark blue and nitrogens in red. Key residues involved in substrate binding are indicated in colors corresponding to their secondary structure regions in (**A**). Polar contacts of NPG with active site waters (red spheres) and residues Arg47, Tyr51 and Ala74 are indicated by yellow dashed lines.

The BM3 heme domain was among the first P450 crystal structures to be published, shedding light on important motifs and residues within the protein (PDB code: 2HPD) [15]. BM3 exhibits a structural integrity conserved across the P450 superfamily, specifically within the B’/C loop, the C- and E-helices, the E/F loop, the β5-sheet and the I-helix [16]. The heme domain of BM3 is relatively flexible and stable and contains the heme prosthetic group necessary for catalysis [17]. BM3 also contains a long, hydrophobic, non-aromatic substrate access channel, which is hypothesized to exist in a state of equilibrium between the substrate-free and substrate-bound forms [18,19]. To date, the intact structure of BM3 has yet to be solved to a higher resolution, most likely due to the dynamic nature of the reductase domain. Figure 1 illustrates the secondary structure of the BM3 heme domain in complex with NPG (N-palmitoyl glycine), highlighting the key residues that play roles in the substrate binding. 

**Figure 2:**
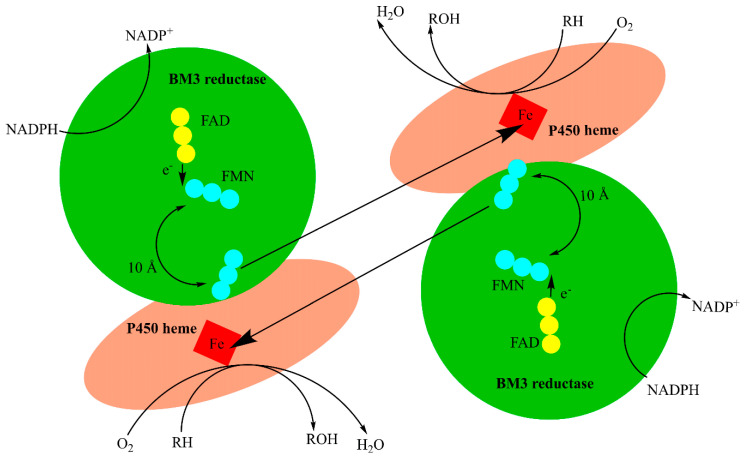
Hypothetical dimeric BM3 electron transfer. The flavin cofactors, FAD and FMN, facilitate electron transfer from the reductase domain to the heme domain. FAD (yellow circles) transfers electrons to FMN (blue circles, which then undergoes a structural rearrangement to facilitate electron transfer to the heme iron (red square).

As mentioned above, BM3 catalysis is an electron-dependent process requiring two electrons delivered from the flavin-containing reductase domain that is attached to the heme domain via the linker region. This fusion yields a highly efficient system with significantly increased activity relative to other monooxygenase P450s. This efficiency enhancement is attributed to the redox partner’s proximity to the heme iron. To date BM3 has the highest reported catalytic activity of any known monooxygenase [20]. Electrons are shuttled in a similar way to the process observed in the microsomal cytochrome P450 reductase (CPR or POR). This transfer starts with the NADPH hydride donation and is followed by a series of FAD and FMN radical oxidations that lead to the electron transfer to the heme domain as a part of the catalytic cycle as shown in Figure 2 [21,22]. The studies with inactive heme and reductase domains suggest that the electrons are shuttled in an intermolecular manner from the FMN in the reductase domain to the heme irons in either heme domain [23]. This rapid electron transfer and malleable active site made BM3 an attractive tool for biotechnological uses.

There are several key residues in BM3 that play a critical role in the structure and function of both the heme and reductase domains, which over the years have made them attractive sites for mutagenesis. For example, to shift the specificity of BM3 to a structurally diverse range of organic substrates or to investigate their structural and mechanistic properties. Figure 1 illustrates the interactions of several of these key residues in complex with NPG. Within the heme domain, Arg47 and Tyr51 sit at the mouth of the substrate access channel and form a hydrophilic binding region that holds fatty acid substrates in place via the carboxylate group [24]. Phe42 provides a ‘cap’ to the substrate access channel in the substrate-bound conformation that is important for catalytic efficiency [15,25]. Phe87 is located near the heme iron in the substrate access channel and plays a role in displacing the axial water molecule which triggers the active site reorganization. It has been demonstrated that Phe87 is a residue of great importance in controlling selectivity and specificity [24]. Other active site residues that have been identified as desirable mutagenesis sites for substrate diversification are Ser72, Val78, Ala82 and Leu188, which play key roles in active site conformations [26]. Glu267 and Thr268 are conserved residues near the kink region of the I-helix (Figure 1, in yellow) and play key roles in proton delivery, oxygen activation and the stabilization of several catalytic cycle intermediates [27,28]. Ala264 is a residue on the I-helix that is postulated to draw the axially coordinated water ligand from the binding site upon the I-helix rearrangement which is typical of substrate binding [29]. Whereas the highly conserved Phe393 residue plays a key role in oxygen binding [30,31]. 

## 2. Wild Type BM3 and Pharmaceutical Metabolism

**Figure 3:**
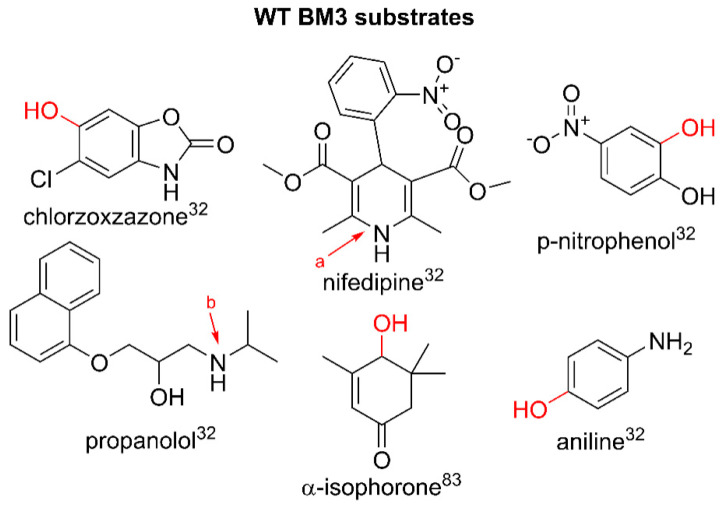
Substrates metabolized by WT BM3. Parent compound structures are in black. Sites of modification are indicated in red: a. aromatization of nifedipine to yield dehydronifedipine, b. dealkylation of propranolol. Literature references are in superscript.

Whilst the wild-type enzyme has a comparatively narrow active site channel and reluctantly binds small molecules that deviate from its hypothesized mid-chain-length, fatty-acid native substrates, there are examples that show the metabolism of drug-like precursors and pharmaceuticals. The studies on WT BM3 have shown that the enzyme can accept substrates metabolized by human P450 isoforms, including catalyzing the hydroxylation of structurally diverse compounds such as chlorzoxazone, aniline and *p*-nitrophenol, the N-dealkylation of propranolol and the dehydrogenation of nifedipine. The V_max_ for all drugs was either comparable or higher than their human P450 counterparts and it was found that WT BM3 exhibited product profiles similar to human CYP3A4 and CYP2E1 [32]. Other examples of WT BM3 metabolizing drug-like compounds show a relatively low activity in comparison to engineered variants designed through iterative mutagenesis studies, including the vitamin A precursor β-ionone, 12-p-nitrophenoxy-carboxylic acid and propylbenzene [33,34,35]. Figure 3 illustrates the modifications on selected drug substrates by WT BM3.

## 3. Engineering BM3 for Drug Metabolite Production

Due to the structural similarity of the BM3 heme domain to the human drug-metabolizing P450s, coupled with the enzyme’s high catalytic efficiency and malleable active site, this domain is an attractive target for mutagenesis studies with the aim of producing pharmaceutical metabolites. The following section aims to give a comprehensive overview of BM3 engineering for the production of valuable drug metabolites. 

### 3.1. Key Promiscuous BM3 Variants and Library Screening 

In early studies, BM3 was targeted as a potential bacterial homologue to many human drug-metabolizing P450s with the aim of shifting the specificity for the production of large quantities of metabolites in an efficient and cost-effective manner. The iterative mutagenesis studies on key active site residues have led to the identification of BM3 variants that exhibit substrate promiscuity. Some of the earliest directed evolution studies on BM3 in Frances Arnold’s group led to the design of the promiscuous variants, 9-10A and 139-3 (see Table 1 for details), which have since been carried forward in subsequent studies on pharmaceutical metabolite production for classes such as steroids, alkaloids, and monosaccharides [36,37,38]. Promiscuous variants were designed based on the triple mutant LVQ in Nico Vermeulen’s group through iterations of error-prone PCR and were shown to exhibit activities up to 70-fold higher than human P450s for clozapine, diclofenac, and acetaminophen [39]. M11 also showed a 200-fold increase in the turnover rate towards dextromethorphan and 3,4-methylenedioxymethylamphetamine compared to WT BM3 and a 90-fold increase compared to human CYP2D6 [40]. The triple mutant GVQ, identified by saturation mutagenesis studies in the Urlacher and Commandeur groups, was found to hydroxylate the omega positions of branched fatty acid derivatives. Further studies demonstrated the ability of GVQ to metabolize structurally diverse human P450 substrates [41,42]. In the above examples of promiscuous variants, there is a clear trend of the introduction of the same or similar point mutations which expand BM3′s catalytic repertoire, including: the neutralization of the polar binding mouth of the active site granting access to non-polar substrates (R47L/Y51F), the addition of polar residues to counteract an overly non-polar environment (L188Q), the removal of Phe87 steric hindrance (F87A/V/L/G) and active site reorganizations (A82L/F, A74E, V78L/F). These residues are often utilized as initial mutagenesis targets in studies aiming to shift the specificity of BM3 to non-natural substrates.

Following the above studies, BM3 libraries were designed with the aim of reaching the promiscuity of the major human drug-metabolizing P450s. One of the first reported libraries was designed by the Arnold group and consisted of point mutations and homologue chimeras. Several variants produced metabolites for verapamil, astemizole and LY294002. A trend in variants evolved from 9-10A and point mutations at Phe87, Ala82 and Val78 exhibited the highest promiscuity and conversion rate. Bulkier substitutions at Ala82 and Phe87 displayed higher conversions but decreased metabolite selectivity [36,43]. A library based on M11 was tested for the O-dealkylation activity of allyloxy-resorufins, with active variants carried forward for affinity testing against several structurally diverse drugs. The variants displayed differing degrees of affinity and selectivity to every drug, with key metabolites produced for buspirone [40,44]. Table 1 gives an overview of the promiscuous variants and their corresponding substrates.

### 3.2. Production of Human P450 Metabolites

Health governing bodies legally require a full drug metabolism and pharmacokinetic profile of lead drug candidates for progression to phase I clinical trials [58]. This profile requires the full structural elucidation of drug metabolites produced at 10% or more of the level of the parent compound, as well as the determination of the in vivo effects [59]. Due to the relatively slow turnover rates of human P450s and the challenges that arise with expressing and purifying membrane-bound proteins, it can be a laborious task to produce metabolites in an easy and cost-efficient manner. BM3′s catalytic similarity to major human P450 drug-metabolizing isoforms and its relative ease of use has made the enzyme an attractive target for mutagenesis studies aiming to shift specificity towards the production of human drug metabolites.

Previously, key variants were described that were designed for the expansion of BM3′s catalytic repertoire for the metabolism of structurally diverse pharmaceuticals. With this in mind, there have been several studies which attempted to design BM3 variants that ‘mimic’ the action of major human drug-metabolizing P450s. The CYP1A2 substrates, phenacetin, ethoxyresorufin and methoxyresorufin, were selectively metabolized into the human metabolites, acetaminophen and resorufin, respectively, by BM3 variants containing combinations of mutations R47L, L86I, F87V and L188Q with an up to three-fold increase in metabolism in comparison to CYP1A2 [60]. The variants containing L75R increase the B’-helix flexibility and BM3’s ability to accept the typically acidic substrates of CYP2C9 and yield human metabolites of ibuprofen and naproxen [61]. A251G/Q307H also mimics the actions of CYP2C9, selectively producing the human metabolites, 4-hydroxydiclofenac, 2-hydroxyibuprofen and 4-hydroxytolbutamide. Structural studies revealed that A251G breaks a salt bridge between Asp251 and Lys221, increasing flexibility and triggering structural rearrangements leading to the rotation of Phe87 [62,63]. A panel of variants that aimed to mimic several isoforms of human drug-metabolizing P450s was designed that led to the production of human drug metabolites for the isoforms CYP2C9 (diclofenac and naproxen); CYP1A2 and CYP2E1 (chlorzoxazone); and CYP3A4 (testosterone, lidocaine and amitriptyline) [64]. Whilst the above variants have the potential to be used as an effective alternative tool for human metabolite production, there are limitations for their use as a replacement for human P450s as they may not exhibit the same metabolic profile. However, their promiscuity can be utilized for the production of human drug metabolites that have been previously identified as crucial for pre-clinical testing.

**Figure 4:**
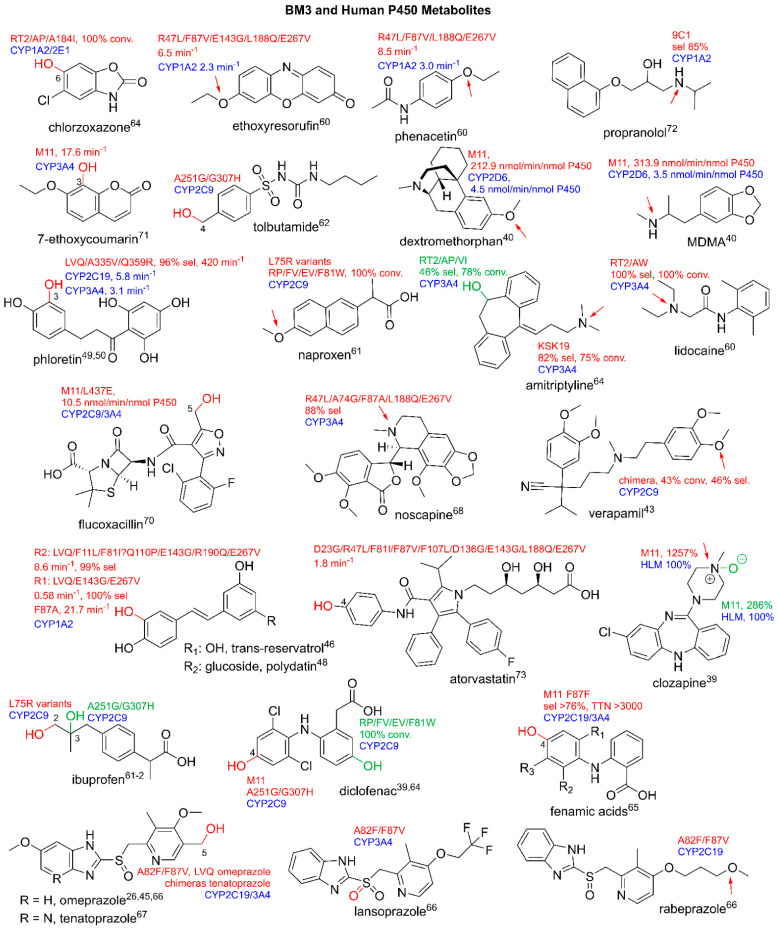
Human P450 and BM3 metabolites. Parent compounds are in black. Sites of modifications are in red and green. Human P450 isoforms are in blue. All arrows indicate the site of N- or O-dealkylation. Conv stands for conversion, Sel stands for selectivity. Rates are in min^−1^, total turnover number (TTN), nmol/min/nmol P450 and relative percentages. Literature references are in superscript.

BM3 variants are shown to target many drugs from the non-steroidal, anti-inflammatory drug class (NSAIDs), for which human metabolites of naproxen, ibuprofen and diclofenac were identified [39,61]. Further studies showed that CYP2C9 and CYP1A2 metabolites of fenamic acids were produced with a high selectivity by BM3 variants and exhibited higher turnover rates in comparison to human liver microsomes [65]. Several drugs in the proton pump inhibitor class are metabolized by BM3 variants, yielding human metabolites. The ‘gatekeeper’ variant, A82F/F87V, for which A82F ‘locks’ the active site in an open conformation and F87V removes a steric hindrance from the active site, produces human metabolites of omeprazole [26,45,66]. Other proton pump inhibitor metabolites are illustrated in Figure 4 [66,67].

**Figure 5:**
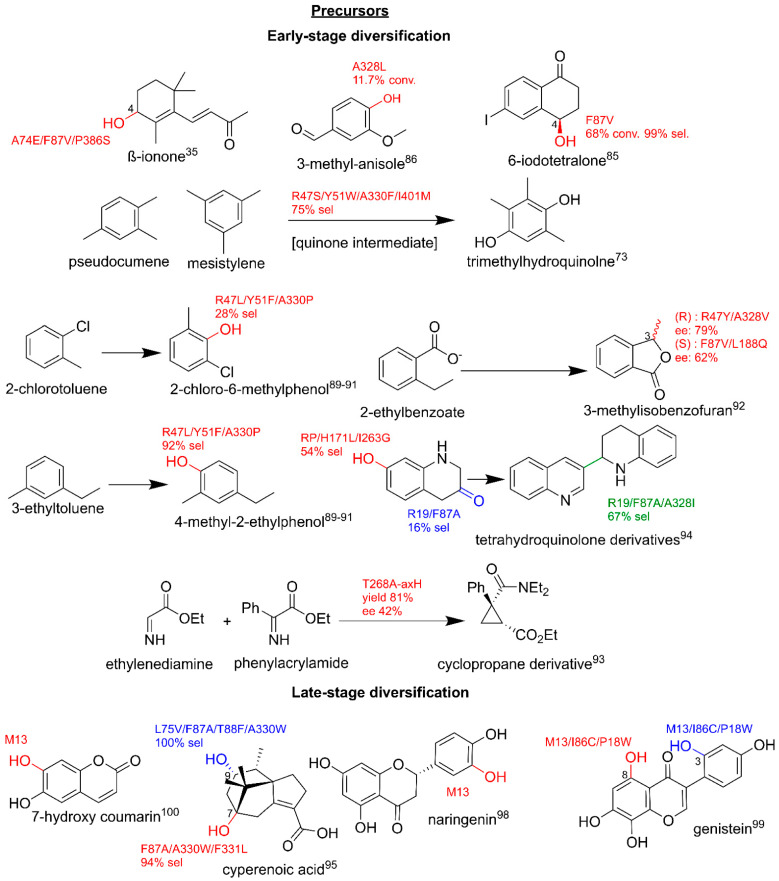
BM3-mediated pharmaceutical precursor production. Parent compounds are in black. Modifications are in red, blue and green with related variants that target the compounds. Conv, conversion; sel, selectivity; ee, enantiomeric excess. Literature references are in superscript.

Steroids are a major pharmaceutical class that are mainly metabolized by CYP3A isoforms. There are many examples in the literature of the production of human steroid metabolites by variants of BM3. The mutations of interest include S72I, which is highly enantioselective for α-hydroxylations, and A82W, which appears to increase P450 coupling efficiency [39,40,52,53,55,56]. Figure 5 illustrates in detail the metabolism of steroids to produce both human and non-human metabolites. There are many more examples of human metabolite production by BM3 variants, which include, but are not limited to, anti-cancer agents such as noscapine, beta blockers such as propranolol and antibiotics such as flucloxacillin [49,50,68,69,70,71,72,73]. BM3-derived human metabolites are illustrated in Figure 4. 

The Arnold group designed some of the first reported BM3 chimeras, where the BM3 heme domain was combined with the homologues, CYP102A2 and CYP102A3, as part of a promiscuous library which aimed to expand the catalytic repertoire for the metabolism of structurally diverse compounds [43]. Attempts have also been made to harness the BM3 reductase domain to facilitate the rapid electron transfer to typically slow drug-metabolizing human P450s for the improved production of human metabolites. Constructs have been designed that link the reductase domain to human P450s for the creation of a self-sufficient, chimeric, human–bacterial, P450-containing system. 

Human P450 chimeras display an advantage over BM3 variants for human metabolite production as, not only is there a typical increase in the electron transfer to the human P450 heme iron leading to increased metabolite production, but a self-sufficient soluble chimera is also easier to work with. Additionally, the same human P450 metabolic profile that is observed in vivo is also retained. This allows the chimeras to be used for metabolite identification in pre-clinical studies. The fusion of the N-terminus CYP2E1 to the C-terminus of the BM3 reductase domain resulted in an active system which showed the formation of human p-nitrophenol and chlorzoxazone metabolites [74]. CYP2C9, CYP2C19 and CYP3A4 constructs were designed with a fusion to the reductase domain and were expressed in *E. coli* to provide a self-sufficient P450 system without the addition of accessory proteins, detergents or phospholipids for activity. All chimeras displayed a 15–30% coupling efficiency and mimicked the WT BM3 dimeric formation in solution, as well as displaying an activity with the FDA-approved probe substrates diclofenac, omeprazole and erythromycin for each isoform, respectively. In addition, the turnover rate was comparable to previously reported results for each human isoform in various reconstituted preparations [75]. The CYP3A4-BM3 reductase domain chimera further displayed an inhibition potential with grapefruit juice, curcumin, and resveratrol, making it a desirable tool for the studies of drug–drug interactions [76]. 

Further studies investigated the role of the heme domain-reductase domain connecting loop on electron transfer efficiency by addition of glycine residues. The data showed that the addition of five glycines increased the V_max_ by two-fold for testosterone [77,78]. BM3’s reductase domain was also fused to the bacterial P450 TxtE, which was shown to be crucial in the thaxtomin pathway; a fusion with 14 amino acid linkers was found to reach a 100% turnover by 8 h with a coupling efficiency 2.2 times higher than standalone TxtE [79]. The success of the BM3 reductase domain chimeras is in part due to the availability of a naturally designed linker region which provides ease of construct design. The recent attempts to design self-sufficient human P450-P450 reductase systems with human CPR are yet to reach the electron transfer rates of BM3 chimeras [80].

### 3.3. Production of Clinically Relevant Pharmaceuticals and Related Precursors

#### 3.3.1. Pharmaceutical Intermediate and Final Product Production

In current research there have been considerable efforts made towards pushing pharmaceutical production, away from traditional organic synthesis routes and towards more economically and environmentally sustainable biosynthesis pathways. There have been several examples of biosynthetic routes in recent years where enzymes were utilized for the production of valuable precursors and final products due to their relatively high yields, low carbon footprint and ability to modify functional groups that were laborious to synthesize chemically [81]. The presence of P450s in many natural product cascades led to the superfamily being of particular interest in the design of engineered biosynthetic pharmaceutical cascades due to their selectivity and their ability to accept small organic compounds and to perform selective hydroxylations which could be used as a molecular handle or for late-stage functionalization [82]. 

There are several key examples of the BM3-mediated biotransformation of pharmaceutical intermediates. The studies that target alkane-moieties, which are typically cyclohexane derivatives, are of particular interest as specific hydroxylations can yield crucial pharmaceutical precursors. The GVQ variant selectively metabolizes branched fatty acids at omega positions forming precursors to macrolide antibiotics [41]. WT BM3 stereoselectively hydroxylates α-isophorone to the vitamin E intermediate, 4-hydroxy-α-isophorone, on a kilogram scale (detailed later in this review). Further studies showed that the F87W/Y86F/L244A/V247L modification improved the selectivity by >98% [83,84]. The metabolite *(R)*-4-hydroxy-iodotetralone, a key intermediate used in palladium-mediated arylations and carbonylations, was produced by F87V at a 99% selectivity [85]. The carotene precursor β-ionone can also be selectively hydroxylated by A74E/F87V/P386S to 4-hydroxy-β-ionone [35].

BM3 variants have also been shown to target a range of benzene derivatives. The production of vanillin from 3-methylanisole was achieved in a whole-cell *E. coli* system via a three-step cascade using the BM3 variant A328L combined with mutated vanillyl alcohol oxidase, albeit at a low conversion of 11.7% and a yield of 1.1% [86]. WT BM3 and the selected variants also selectively hydroxylate the methyl-substituted benzene derivatives, pseudocumene and mesitylene, to the phenolic building blocks used in the α-tocopherol synthesis via quinolone intermediates. For example, the introduction of the point mutation A330F increased the selectivity towards metabolite trimethylhydroquinolone to 75% [87,88]. The selective hydroxylation of various halogenated alkyl benzene derivatives by R47L/Y51F/A330P yields key intermediates such as 2-chlorotoluene and 4-methyl-2-ethylphenol with turnovers of 500 to 1600 min^−1^. Interestingly, ortho-substituted toluenes displayed group migrations of the methyl group to meta-positions due to a hypothesized epoxidation, ring opening and migration [89,90,91]. The stereoselective hydroxylation of 2-alkylated benzoic acid esters to produce phthalide (fungicide intermediates) and isocoumarin (serine protease inhibitors) derivatives was trialed on a preparative scale, which led to the hydroxylation of 2-ethylbenzoate to enantiomers of 3-methylisobenzofuran [92]. 

The cyclopropanation of phenylacrylamide is the first step in a semi-synthetic route to the antidepressant levomilinacipran. This cyclopropanation reaction was achieved by replacing the axially heme iron coordinated cysteine with histidine (C400H) and by the introduction of the point mutation T268A. In this variant the enzyme no longer proceeds through the canonical mechanism utilizing an iron-oxo intermediate described earlier, but now via a carbene transfer route [93]. A 48-variant library was screened for the production of tetrahydroquinolone derivatives, yielding diverse products with varying degrees of selectivity and functional group modifications. The specific mutations appeared to control the type of biotransformation and led to the expansion beyond BM3’s typical hydroxylations, including A328L which played a role in the metabolite dimer formation. F87A/V, A328I and I263G favored C5 and C6 oxidations and also promoted metabolite dimer formation, with F87 variants displaying activity towards aromatization. The range of biotransformations performed by different variants in this study highlighted BM3’s role as a selective multi-functional oxidase [94].

**Figure 6:**
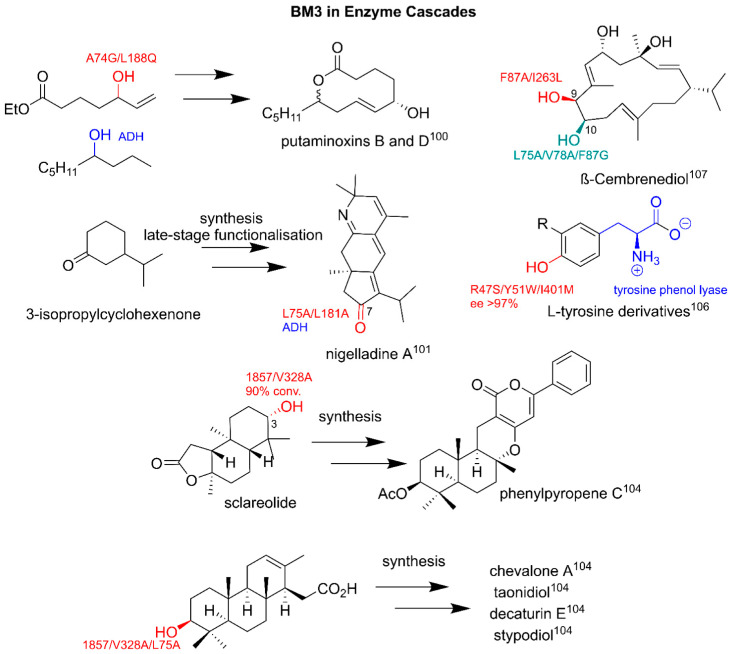
BM3 in enzyme cascades. Parent compounds are in black. BM3-related modifications are in red and green. Other enzymes are in blue. Conv, conversion; sel, selectivity; ee, enantiomeric excess. Literature references are in superscript.

There are also key examples of utilizing BM3 variants for selective, late-stage oxy-functionalization to yield clinically relevant pharmaceuticals. A late-stage hydroxylation by P450s is advantageous for the addition of functional groups that are chemically laborious to synthesize and removes the need for protecting groups on typically reactive hydroxyl groups. The preparative scale production of cyperenoic acid metabolites gives high yields and high selectivity for products that display antiangiogenic-promoting effects [95]. Glucocorticosteroids are typically steroid-derived C16 alcohols, which require selective hydroxylation; the combinations of BM3 variants at R47, Y51 and F87, displayed selectivities of above 90% (Figure 6) [96]. M13 variant was utilized for the specific hydroxylations to yield derivatives of existing pharmaceuticals that displayed enhanced medicinal properties [71]. This variant was also employed for the production of the anticancer and antibacterial agent esculetin on a preparative 3L scale, as well as for synthesizing the more potent flavonoid antibacterial and anticancer agent, eriodictyol. The additional mutations, I86C/P18W, led to the production of 3′-hydroxygenistein and 8-hydroxygenestein, which displayed more potent pharmaceutical effects than the parent compound and the angiogenesis inhibitor genistein [97,98,99].

#### 3.3.2. BM3 in Semi-Synthetic Routes and Biosynthetic Cascade Production of Pharmaceuticals

As demonstrated previously, BM3 and its extensive catalytic repertoire make the enzyme an attractive biotechnological tool for the incorporation into semi-synthetic routes and biosynthetic enzyme cascades for the production of clinically relevant pharmaceuticals. There are many examples of utilizing BM3 for typically chemically laborious hydroxylations; as either a molecular handle for functional group additions or for late-stage diversification. The cascade synthesis of the antifungal putaminoxins, B and D, utilized A74G/L188Q in tandem with alcohol dehydrogenase (ADH) for hydroxylation and allylic alcohol formation, which was a key step in macrolactone formation [100]. 

Another example of incorporating BM3 into a semi-synthetic route is the synthesis of nigelladine A. Starting with 3-isopropylcyclohexenone, a chemical synthesis route to the intermediate tricycle was achieved with a singular allylic oxidation required to achieve nigelladine A. The final oxidation and imine bond formation was performed by L75A/L181A and ADH at a 43% yield [101]. F87V and WT BM3, respectively, can selectively epoxidate arachidonic and linoleic acid to produce 14-*(S)*-,15-*(R)*-epoxyeicosatrienoic acid and leukotoxin B, with approximately a 45% conversion on a mg scale, which was carried forward to produce 14-*(R)*-,15-*(S)*-epoxyeicosatrienoic acid [102]. 

The production of the anti-cancer and antimicrobial meroterpenoids was achieved via the design of modular retrosynthetic pathways. The addition of molecular handle hydroxyl groups was achieved by variants related to 1857 (V78A/A82G/F87V/P142S/T175I/A184V/F205C/S226R/H236Q/E252G/R255S/A290V/L353V) [103,104]. The selective 3-hydroxylation of sclareolide, an antifungal precursor to arisugacin F and phenylpyropene C, gave an 80–90% conversion and a 60–70% yield for 1857/V328A. A 62% yield was achieved by 1857/V328A/L75A for the oxidation of a tricyclic intermediate at the C3 position, forming a key intermediate for further chemical modification, ultimately yielding either chevalone or stypodiol [104]. 

The synthesis of L-tyrosine derivatives, which were intermediates to anticancer drugs such as saframycin A, was achieved via the R47S/Y51W/I401M-mediated ortho-hydroxylation of monosubstituted benzenes in a one-pot, two-step synthesis. The subsequent addition of pyruvate and ammonia, utilizing a variant of tyrosine phenol lyase, led to hydroxy-derivatives of tyrosine, with the ortho-hydroxylation leading to tyrosine’s distinctive para-phenol [105]. On a preparative scale, low-to-medium turnover rates were observed with a selectivity above 50% and enantiomeric excesses of >97% for all derivatives [106]. BM3 could also be used as a multi-step mixed-function oxidase. A two-step hydroxylation of the macrocyclic diterpenoid β-cembrenediol by BM3 variants yielded (9*S*,10*S*)-β-cembrenetetraol, displaying up to 90% enantiomeric excess [107]. Figure 6 illustrates key examples of BM3 in enzyme cascades.

### 3.4. Utilizing BM3 for Production of Emerging Pharmaceuticals

**Figure 7:**
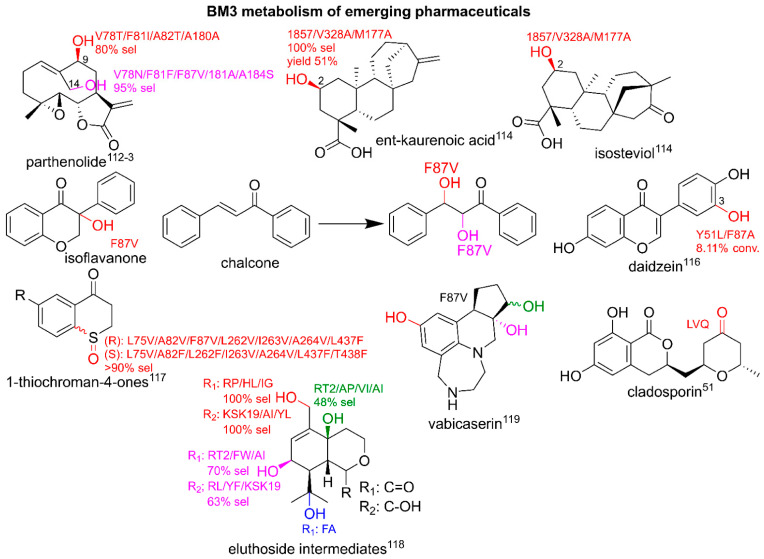
BM3-mediated metabolism of emerging pharmaceuticals. Parent compounds are in black. Modifications are in red, pink, green and blue. Conv, conversion; sel, selectivity. Literature references are in superscript.

The research into the biosynthetic production of pharmaceuticals has led to harnessing and engineering enzymes from natural product cascades for a generation of emerging pharmaceuticals to explore chemical space that is difficult to reach by means of chemical synthesis [108]. As detailed previously, the catalytic repertoire of BM3 extends beyond what is typically available in traditional synthesis routes, and there are many examples of the production of analogues resulting from BM3-mediated oxidation modifications not previously observed. This section gives an overview of the utilization of BM3 for the production of emerging pharmaceuticals and is summarized in Figure 7. 

Previously, the production of BM3 and human steroid metabolites was discussed. There were also extensive studies into the hydroxylation of steroid positions that were difficult to reach for late-stage diversification or as molecular handles. The variant, 139-3 (see Table 1), hydroxylates androstenedione at the challenging 1α-position. This interesting reaction is postulated to be caused by Arg379 which is thought to play a role in active site orientation [58]. Steroidal C6β- and C7β-alcohols are shown to display neuroprotective and anti-inflammatory properties but are difficult to chemically oxidate. Several variants are shown to hydroxylate at these positions with high selectivities [109,110]. Table 2 gives an overview of BM3-mediated steroid hydroxylation.

Terpenoids are attractive targets for BM3 diversification. The sesquiterpene lactone parthenolide was previously shown to possess anti-cancer activity but requires the improvement of pharmacokinetic properties. The BM3-mediated addition of hydroxyl groups as molecular handles at the C9- and C14- allowed the addition of the aromatic functional groups and led to the discovery of potent parthenolide-based antileukemic agents [111,112]. Studies also aimed to diversify meroterpenoids; selective hydroxylations at the C2-positions of *ent*-kaurenoic acid and a derivative of isosteviol led to the production of cochleareine and spiramilactone C derivatives [113]. 

The diversification of the flavonoid class of pharmaceuticals was explored using variants of BM3 as they displayed a wide range of medicinal properties. The flavanone derivative, s chalcone and isoflavone, were hydroxylated in multiple positions by F87V, with dihydroxyflavanone derivatives displaying the highest IC_50_ values for antioxidant activity via testing the lipid peroxidation in rat brain homogenate [114]. Further studies showed that the antioxidant daidzein was turned over by Y51L/F87A to 3-hydroxydaidzein, a compound with more potent effects [115].

The diversification of building blocks for pharmaceuticals is also of interest as the molecular handle of the hydroxyl groups can lead to a wide range of new analogues. The sulfoxidation of 1-thiochroman-4-ones can lead to building blocks of pharmaceutical interest with potential anti-cancer properties. BM3 was engineered for *(S)*- and *(R)*-sulfoxidation as a replacement to chiral metal catalysts with high selectivities for specific variants displayed [116]. Other key examples of the BM3-mediated diversification of pharmaceuticals include the stereoselective hydroxylation of eleuthoside intermediates, related to the emerging anti-cancer agent, eleuthorobin.

From a panel of BM3 variants including GVQ, the hydroxylation of cyclohexane-based moieties produced a range of oxidized metabolites with high conversion rates and varying degrees of selectivity for several positions. Phe87 variants displayed the ability to further oxidize alcohols to aldehydes with a high regioselectivity and preparative scale reactions showed the ability of BM3 to produce lead drug candidates under scaled-up conditions [117]. The late-stage functionalization of the antimalarial compound cladosporin by LVQ led to the production of ketone derivatives that expressed some degree of potency in the micromolar range against *P. falciparum* [51]. Late-stage oxidations can present a challenge with selectivity and the production of unwanted by-products. An attempt to solve this issue was trialed with vabicaserin with the addition of a protecting group to ‘steer’ the oxidation site of BM3 variants; an analogue was metabolized by F87V to yield several metabolites with hydroxylations at various positions before the cleavage of the protecting groups [118].

## 4. Stretching the Use of BM3

In addition to the extensive studies on engineering the BM3 heme domain to expand the catalytic repertoire, there are also studies based on other engineering strategies for the production of valuable pharmaceuticals. This section aims to give an overview on how BM3 can be used outside of the traditional directed evolution approaches and how it can be utilized for real-world applications.

### 4.1. Decoy Molecules

Decoy molecules are small molecules that co-occupy active sites without metabolism or inhibition and ‘activate’ enzymes without the need for mutagenesis by occupying space in the active site. The addition of the decoy molecules allows active sites to accept smaller substrates, for example in the WT BM3 heme domain. With the aid of semi-synthetic decoy molecules based on amino acid derivatives, WT BM3 can catalyze the oxidation of benzene to the pharmaceutical precursor phenol, a key intermediate in the synthetic routes of drugs such as aspirin [119,120]. C7-L-Proline-L-Phenylalanine increased the turnover of benzene by WT BM3 from negligible rates to 259 min^−1^, whilst the addition of *N*-heptyl-l-prolyl-l-phenylalanine led to a phenol yield of 59% [120]. C4- to C8-chain length fatty acids were also utilized for the enantioselective hydroxylation of the pharmaceutical precursors styrene, ethylbenzene and thioanisole. The results showed that the C5–C7 chain length displayed a balance of increased turnover and selectivity; *(S)*-styrene oxide was produced with similar selectivities for all decoy molecules with hexanoic acid displaying the highest turnover (334 min^−1^). Ethylbenzene heptanoic acid displayed the best turnover (28 min^−1^) and selectivity (68%) for *(R)*-phenylethan-1-ol [121].

### 4.2. Industrial and Preparative Scale-up

The biocatalytic production of valuable drug metabolites on an industrial scale is desirable, as these processes are often cheaper, greener, and more efficient with higher purity and yields. There are several examples of scaling up BM3 for preparative (mg) and industrial (g to kg) metabolite production, and there are numerous examples of commercialized production. One of the most notable examples from recent years is the stereoselective hydroxylation of α-isophorone to the carotenoid and the vitamin E precursor 4-hydroxy-α-isophorone by WT BM3 on a kilogram scale. WT BM3 was co-expressed with glucose dehydrogenase in *E. coli* and in 1000 L reactors, providing a yield of 0.95 kg of purified oxy-metabolite and a productivity of 1000 mg/L/h. The success of this whole-cell scale-up paves the way for future industrial metabolite production utilizing BM3 and related variants [84]. The production of the anti-tumor drug, desmethyl-colchicine, from colchicine on a 70 L bioreactor scale was achieved by *E. coli* whole-cell reactions expressing WT BM3; ~80% conversion was achieved in 48 h with a productivity of 6.62 mg/L/h, and a maximum yield of 5.96 g/L [122]. Throughout this review there are examples of the preparative scale-up of BM3-mediated metabolite production; this includes but is not limited to 16-β-hydroxy-norandrostenedione, 3-methylbenzofuran, C7-hydroxy-cyperenoic acid, esculetin and leukotoxin B [54,92,95,97,102]. 

### 4.3. Medicinal Usage of BM3

Future emerging therapies are exploring the use of enzymes as in vivo alternatives or enhancements to existing treatments, as they can be harnessed for uses such as in vivo imaging and site-specific, pro-drug metabolism [123]. A key example of utilizing BM3 for in vivo imaging includes the detection of the neurotransmitter dopamine using the BM3 heme domain as a contrast agent for magnetic resonance imaging. The dopamine binding to sites near the paramagnetic heme iron of evolved BM3 variants decreases signal enhancement and a shift in optical absorbance is also observed. The release of dopamine was imaged in PC12 cells and the brains of live animals [124]. Further work led to the development of the evolved BM3 sensors for dopamine (T268A/I263A/T438V/A328G, *K_D_* 1.3 µM) and serotonin (F87L/T438L/T268S/L437Q, *K_D_* 700 nM) which were both utilized for the neurotransmitter release quantitation in vitro [125]. 

Recent studies on the in vivo therapeutic delivery systems encompassed BM3 as part of bionanoparticle systems, with the purpose of harnessing the metabolism for therapeutic gain. Several drugs require activation by the P450 mediated metabolism; however, once activated, the bioavailability may significantly decrease before reaching the therapeutic target due to polar metabolites typically exhibiting shorter half-lives. Delivering BM3 variants to therapeutic sites has been explored with the aim of increasing prodrug potency. 

The activation of the anticancer prodrugs, cyclophosphamide and ifosfamide, by M11 and M11 L437S, identified in previous studies, were found to be highly active for 4-hydroxylation of both prodrugs; M11 displayed catalytic efficiencies of ~10,000 and ~1350 mol product/min/mol CYP/mM for cyclophosphamide and ifosfamide, respectively. The extracellular bioactivation led to a cytotoxicity in U2OS cells [126]. The biotransformation of the endocrine disruptor compounds such as bisphenol A, nonylphenol and triclosan by P450s was crucial to counteract their harmful effects. 

A bionanoreactor encompassing the capsid of the bacteriophage P22 with the peroxygenase variant 21B3 (identified in previous studies), functionalized with glucose oxidase on the outside as part of an electron transfer system, led to a turnover rate of approximately half of the free BM3, making the system a viable route for therapeutic detoxification [127,128]. The usage of BM3 in a bionanoreactor as part of drug delivery was shown when encompassing F87A and glucose oxidase in a nanoreactor crosslinked with human serum albumin, with the aim of improving tamoxifen efficacy. The selective delivery of the nanoreactor to MCF-7 cells was observed showing a triple effect of depleting glucose levels, an antioxidant effect from the glucose–oxidase byproduct, hydrogen peroxide, and the metabolism of prodrug tamoxifen into its active metabolite by F87A. MTT assays showed that the co-administration of tamoxifen and the bionanoreactor decreased cell viability from ~45% to ~10% when compared to the drug alone [129].

## 5. Summary, Conclusions and Outlook

In terms of directed evolution, BM3 is a key focal point and one of the best examples to highlight the advantages of the biosynthetic production of valuable small molecules. The enzyme’s ease of production, catalytic repertoire and ability to perform specific oxidations that are typically chemically laborious makes BM3 one of the most attractive biotechnological tools for current and future metabolite production. Several high-activity, promiscuous variants were developed using a range of engineering strategies. The comparison of these studies, in conjunction with extensive active site investigations, has allowed researchers to pinpoint crucial key residues that play pivotal roles in structurally diverse substrate acceptance, enantio- and regioselectivity, metabolic rates and electron transfer. The malleable nature of the BM3 active site has, therefore, led to the publication of hundreds of papers investigating the metabolite production of a vast range of structurally diverse compounds, of which a significant number have targeted pharmaceuticals. The similarities between major human drug-metabolizing P450s and BM3’s metabolic profiles led to much research focusing on designing human P450 ‘mimics’, or selective variants for the production of human P450 metabolites, as an attractive alternative to the relatively slower and membrane-bound human isoforms. Whilst the BM3 heme domain variants are unlikely to be used as a replacement for human P450s in the pre-clinical metabolic profiling of emerging pharmaceuticals, advances such as the BM3 reductase-human P450 heme chimeras show that there is a potential for using the BM3 flavin domain in pharmacokinetic studies. There are also many examples which show that BM3 has the potential to be harnessed in the production of the pharmaceutical intermediates and final products. The enzyme’s ability to specifically oxidize a range of positions in structurally diverse compound classes with a high selectivity and high yields has not only been demonstrated in the literature, but on preparative and industrial scales which has led to key examples of the commercial production of pharmaceutical intermediates. Its utility as a mixed function oxidase and ease of expression in whole-cell microorganism systems has led to the addition of several key variants to biosynthetic cascades, which are arguably the future of pharmaceutical production. In the current climate, BM3 has the potential to be used as a key biotechnological tool, allowing the shift away from traditional organic synthesis routes towards more sustainable biosynthetic means of production. 

BM3 also has the ability to diversify existing and new pharmaceutical libraries, as its catalytic abilities allow oxidized functional groups to be utilized as molecular handles or for late-stage diversification. Key examples in the literature show the BM3-mediated production of more potent analogues of several emerging pharmaceuticals, solidifying its future role in the enzyme-mediated expansion of the pharmaceutical chemical space that organic synthesis cannot reach. BM3 has also been shown to be a valuable tool in emerging medicinal therapeutics, where the delivery of selected variants to target sites in the bionanoreactors led to successes in targeting different medical issues, such as in vivo imaging and increasing drug efficacy. The literature is filled with examples of BM3 metabolite production with a wide range of purposes, and this promiscuous bacterial P450 will most certainly continue to appear in future academic and industrial settings.

## Figures and Tables

**Table 1:** Promiscuous BM3 variants and their corresponding drug substrates. Asterisks indicate where further point mutations were introduced for metabolism.

BM3 Variant	Point Mutations	Substrates
LVQ [39]	R47L/F87V/L188Q	clozapine [39], diclofenac [39], acetaminophen [39], omeprazole [45], reservatrol * [46], polydatin [47,48], phloretin * [49,50], cladosporin [51]
M01 [39]	LVQ/E267V/G415S/G1049E	clozapine [39], diclofenac [39], acetaminophen [39], testosterone * [52], noresthisterone * [53], methoxyresorufin [44], ethoxyresorufin [44]
M02 [39]	LVQ/L86I/N319T/A964V	clozapine [39], diclofenac [39], acetaminophen [39], norandrostenedione [54], methoxyresorufin [44], ethoxyresorufin [44], buspirone [44], amitriptyline [44], aripiprazole [44]
M05 [39]	LVQ/F81I/E267V/G415S	clozapine [39], diclofenac [39], acetaminophen [39], methoxyresorufin [44], ethoxyresorufin [44]
M11 [39]	LVQ/E64G/F81I/E143G/E267V/G415S	clozapine [39], diclofenac [39], acetaminophen [39], Testosterone * [52,55], norethisterone * [53,55], dextromethorphan [40], nifedipine [40], midazolam [40], 3,4-methylenedioxymethylamphetamine [40], estradiol [14,56], methoxyresorufin [44], ethoxyresorufin [44], buspirone [44], duloxetine [44], thioridazine [44], ondansetron [44], imatinib [44], omeprazole [44], rosiglitazone [44], paroxetine [44], norethisterone [44], resveratrol [44], dihydrobenzophenone [44], 17α-ethinylestradiol [44], bisphenol A [44], diethylstilbestrol [44], hexestrol [44], estriol [44], benzophenone-3 [44], aldrin [44], testosterone [44], tramadol [44], imidazole [44], ketoconazole [44]
9-10A [36]	R47C/V78A/K94I/P142S/T175I/A184V/F205C /S226R/H236Q/E252G/R255S/A290V/L353V	verapamil [43], astemizole [43], LY294002 [43]
139-3 [38]	V78A/H138Y/T175I/V178I/A184V/ H236Q/ E252G/R255S/A290V/A295T/L353V	androstenedione [57]
GVQ [41]	A74G/F87V/L188Q	testosterone [42], amodiaquine [42], dextromethorphan [42], acetaminophen [42], 3,4-methylenedioxymethylamphetamine [42]

**Table 2:** BM3-mediated steroid metabolism. Only sites of modification are identified. * Indicates human CYP3A metabolite. Conv, conversion; sel, selectivity; ee, enantiomeric excess.

Steroid	Structure	OH- Position	Variant	Parameters
Testosterone	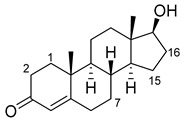	2β *	RLYF/KSK19 * [64]	100% conv, 61% sel
		1β	H171L/Q307H/N319Y/F87A/T260G/P329G/A330W [109]	71% sel, 76% conv
		7β	R47W/S72W/F77Y/V78L/F81I/A82L/T88S/M177T/M185Q/L188Q/I209T [110]	90% sel
		15β *	KSK19/FV/QP [64]	96% sel, 83% conv
		16α *	M11/V87I/S72I [52] M01/A82W/S72I [52]	90% ee 95% ee
		16β *	M01/A82W [52] M11/V87I [52]	100% ee 100% ee
Estradiol	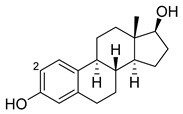	2	M11 [56]	47 min^−1^
Norandrostenedione	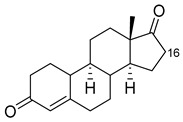	16β *	M02 [54]	95% sel
Nandrolone	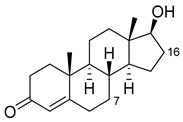	7β	R47W/S72W/F77Y/V78L/F81I/A82L/T88S/M177T/M185Q/L188Q/I209T [110]	75% sel
		16α	R47L/S72I/A82F/F87I/L188C/A330W [96]	98% sel
		16β	R47W/A82W/F87V/L181Q [96]	90% sel
Norethindrone	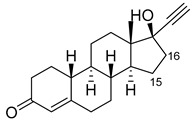	15β *	M11/A82W/V87A [53]	100% sel
		16β *	M01/A82W [53]	96% sel
Boldenone	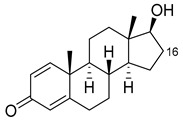	16α	R47L/Y51W/S72I/A82W/F87I/L181C [96]	97% sel
		16β	R47W/A82W/F87V/L181Q [96]	72% sel
Androstenedione	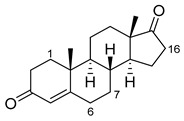	1α	139-3 [57]	
		6β	H171L/Q307H/N319Y/F87V/I263G [109]	78% sel, 29% conv
		7β	R47W/S72W/F77Y/V78L/F81I/A82L/T88S/M177T/M185Q/L188Q/I209T [110] R47L/Y51F/H171L/Q307H/N319Y/ F87A/A184I/T260G/A328G [109]	90% sel 81% sel, 94% conv
		16α	R47W/Y51W/S72I/A82F/F87I/L181C [96]	95% sel
		16β	R47W/Y51H/A82W/F87V/L181Q [96]	100% sel

## References

[1] Roberts A.A., Ryan K.S., Moore B.S., Gulder T.A.M. (2010). Total (Bio)synthesis: Strategies of nature and of chemists. Top. Curr. Chem..

[2] Rupasinghe S., Schuler M.A., Kagawa N., Yuan H., Lei L., Zhao B., Kelly S.L., Waterman M.R., Lamb D.C. (2006). The cytochrome P450 gene family CYP157 does not contain EXXR in the K-helix reducing the absolute Conserved P450 residues to a single cysteine. FEBS Lett..

[3] Guengerich F.P., Macdonald T.L. (1984). Chemical mechanisms of catalysis by cytochromes P-450: A unified view. Acc. Chem. Res..

[4] Whitehouse C.J.C., Bell S.G., Wong L.-L. (2012). P450 BM3 (CYP102A1): Connecting the dots. Chem. Soc. Rev..

[5] Iyanagi T. (2007). Molecular mechanism of phase I and phase II drug-metabolizing enzymes: Implications for detoxification. Int. Rev. Cytol..

[6] Miura Y., Fulco A.J. (1974). (ω-2) Hydroxylation of fatty acids by a soluble system from *Bacillus megaterium*. J. Biol. Chem..

[7] Miura Y., Fulco A.J. (1975). ω-1, ω-2 and ω-3 hydroxylation of long-chain fatty acids, amides and alcohols by a soluble enzyme system from *Bacillus megaterium*. Biochim. Biophys. Acta.

[8] Hare R.S., Fulco A.J. (1975). Carbon monoxide and hydroxymercuribenzoate sensitivity of a fatty acid (ω-2) hydroxylase from *Bacillus megaterium*. Biochem. Biophys. Res. Commun..

[9] Narhi L.O., Fulco A.J. (1987). Identification and Characterization of two functional domains in cytochrome P-450BM-3, a catalytically self-sufficient monooxygenase induced by barbiturates in *Bacillus megaterium*. J. Biol. Chem..

[10] Narhi L.O., Fulco A.J. (1986). Characterization of a catalytically self-sufficient 119,000-dalton Cytochrome P-450 monooxygenase induced by barbiturates in *Bacillus megaterium*. J. Biol. Chem..

[11] Porter T.D. (1991). An unusual yet strongly conserved flavoprotein reductase in bacteria and mammals. Trends Biochem. Sci..

[12] Lewis D.F.V., Watson E., Lake B.G. (1998). Evolution of the cytochrome P450 superfamily: Sequence alignments and pharmacogenetics. Mutat. Res..

[13] Fulco A.J. (1967). Chain elongation, 20hydroxylation, and decarboxylation of long chain fatty acids by yeast. J. Biol. Chem..

[14] English N., Hughes V., Wolf C.R. (1994). Common pathways of cytochrome P450 gene regulation by peroxisome proliferators and barbiturates in *Bacillus megaterium* ATCC14581. J. Biol. Chem..

[15] Ravichandran K.G., Boddupalli S.S., Hasemann C.A., Peterson J.A., Deisenhofer J. (1993). Crystal structure of hemoprotein domain of P450BM-3, a prototype for microsomal P450′s. Science.

[16] Hasemann C.A., Kurumbail R.G., Boddupalli S.S., Peterson J.A., Deisenhofer J. (1995). Structure and function of cytochromes P450: A comparative analysis of three crystal structures. Structure.

[17] Anzenbacher P., Hudeček J. (2001). Differences in flexibility of active sites of cytochromes P450 probed by resonance raman and UV-Vis absorption spectroscopy. J. Inorg. Biochem..

[18] Li H., Poulos T.L. (1995). Modeling protein–substrate interactions in the heme domain of cytochrome P450BM−3. Acta Crystallogr. Sect. D Biol. Crystallogr..

[19] Joyce M.G., Girvan H.M., Munro A.W., Leys D. (2004). A single mutation in cytochrome P450 BM3 induces the conformational rearrangement seen upon substrate binding in the wild-type Enzyme. J. Biol. Chem..

[20] Daff S.N., Chapman S.K., Turner K.L., Holt R.A., Govindaraj S., Poulos T.L., Munro A.W. (1997). Redox control of the catalytic cycle of flavocytochrome P-450 BM3. Biochemistry.

[21] Gonvindaraj S., Li H., Poulos T.L. (1994). Flavin supported fatty acid oxidation by the heme domain of *Bacillus megaterium* cytochrome P450BM-3. Biochem. Biophys. Res. Commun..

[22] Sevrioukova I.F., Peterson J.A. (1995). Reaction of carbon-monoxide and molecular-Oxygen with P450terp (CYP108) and P450BM-3 (CYP102). Arch. Biochem. Biophys..

[23] Neeli R., Girvan H.M., Lawrence A., Warren M.J., Leys D., Scrutton N.S., Munro A.W. (2005). The dimeric form of flavocytochrome P450 BM3 is catalytically functional as a fatty acid hydroxylase. FEBS Lett..

[24] Li H., Poulos T.L. (1997). The structure of the cytochrome P450BM-3 haem domain complexed with the fatty acid substrate, palmitoleic acid. Nat. Struct. Biol..

[25] Noble M.A., Miles C.S., Chapman S.K., Lysek D.A., Mackay A.C., Reid G.A., Hanzlik R.P., Munro A.W. (1999). Roles of key active-site residues in flavocytochrome P450 BM3. Biochem. J..

[26] Butler C.F., Peet C., Mason A.E., Voice M.W., Leys D., Munro A.W. (2013). Key mutations alter the cytochrome P450 BM3 conformational landscape and remove inherent substrate bias. J. Biol. Chem..

[27] Volz T.J., Rock D.A., Jones J.P. (2002). Evidence for two different active oxygen species in cytochrome P450 BM3 mediated sulfoxidation and N-Dealkylation Reactions. J. Am. Chem. Soc..

[28] Cryle M.J., De Voss J.J. (2006). Is the ferric hydroperoxy species responsible for sulfur oxidation in cytochrome P450s?. Angew. Chemie Int. Ed..

[29] Haines D.C., Tomchick D.R., Machius M., Peterson J.A. (2001). Pivotal role of water in the mechanism of P450BM-3. Biochemistry.

[30] Ost T.W.B., Clark J., Mowat C.G., Miles C.S., Walkinshaw M.D., Reid G.A., Chapman S.K., Daff S. (2003). Oxygen activation and electron transfer in flavocytochrome P450 BM3. J. Am. Chem. Soc..

[31] Ost T.W.B., Miles C.S., Munro A.W., Murdoch J., Reid G.A., Chapman S.K. (2001). Phenylalanine 393 exerts thermodynamic control over the heme of flavocytochrome P450 BM3. Biochemistry.

[32] Di Nardo G., Fantuzzi A., Sideri A., Panicco P., Sassone C., Giunta C., Gilardi G. (2007). Wild-type CYP102A1 as a biocatalyst: Turnover of drugs usually metabolised by human liver enzymes. J. Biol. Inorg. Chem..

[33] Whitehouse C.J.C., Yang W., Yorke J.A., Tufton H.G., Ogilvie L.C.I., Bell S.G., Zhou W., Bartlam M., Rao Z., Wong L.L. (2011). Structure, electronic properties and catalytic behaviour of an activity-Enhancing CYP102A1 (P450 BM3) Variant. Dalt. Trans..

[34] Li Q.S., Schwaneberg U., Fischer M., Schmitt J., Pleiss J., Lutz-Wahl S., Schmid R.D. (2001). Rational evolution of a medium chain-specific cytochrome P-450 BM-3 variant. Biochim. Biophys. Acta Protein Struct. Mol. Enzymol..

[35] Urlacher V.B., Makhsumkhanov A., Schmid R.D. (2006). Biotransformation of β-ionone by engineered cytochrome P450 BM-3. Appl. Microbiol. Biotechnol..

[36] Peters M.W., Meinhold P., Glieder A., Arnold F.H. (2003). Regio- and enantioselective alkane hydroxylation with engineered cytochromes P450 BM-3. J. Am. Chem. Soc..

[37] Lewis J.C., Mantovani S.M., Fu Y., Snow C.D., Komor R.S., Wong C.-H., Arnold F.H. (2010). Combinatorial alanine substitution enables rapid optimization of cytochrome P450BM3 for selective hydroxylation of large substrates. ChemBioChem.

[38] Glieder A., Farinas E.T., Arnold F.H. (2002). Laboratory evolution of a soluble, self-sufficient, highly active alkane hydroxylase. Nat. Biotechnol..

[39] Damsten M.C., van Vugt-Lussenburg B.M.A., Zeldenthuis T., de Vlieger J.S.B., Commandeur J.N.M., Vermeulen N.P.E. (2008). Application of drug metabolising mutants of cytochrome P450 BM3 (CYP102A1) as biocatalysts for the generation of reactive metabolites. Chem. Biol. Interact..

[40] Van Vugt-Lussenburg B.M.A., Stjernschantz E., Lastdrager J., Oostenbrink C., Vermeulen N.P.E., Commandeur J.N.M. (2007). Identification of critical residues in novel drug metabolizing mutants of Cytochrome P450 BM3 using random mutagenesis. J. Med. Chem..

[41] Budde M., Morr M., Schmid R.D., Urlacher V.B. (2006). Selective Hydroxylation of highly branched fatty acids and their derivatives by CYP102A1 from *Bacillus megaterium*. ChemBioChem.

[42] Vugt-Lussenburg B.M.A., Damsten M.C., Maasdijk D.M., Vermeulen N.P.E., Commandeur J.N.M. (2006). Heterotropic and homotropic cooperativity by a drug-metabolising mutant of cytochrome P450 BM3. Biochem. Biophys. Res. Commun..

[43] Sawayama A.M., Chen M.M.Y., Kulanthaivel P., Kuo M.-S., Hemmerle H., Arnold F.H. (2009). A panel of cytochrome P450 BM3 variants to produce drug metabolites and diversify lead compounds. Chem. A Eur. J..

[44] Reinen J., Ferman S., Vottero E., Vermeulen N.P.E., Commandeur J.N.M. (2011). Application of a fluorescence-based continuous-flow bioassay to screen for diversity of cytochrome P450 BM3 mutant libraries. J. Biomol. Screen..

[45] Ryu S.H., Park B.Y., Kim S.Y., Park S.H., Jung H.J., Park M., Park K.D., Ahn T., Kang H.S., Yun C.H. (2014). Regioselective hydroxylation of omeprazole enantiomers by bacterial CYP102A1 mutants. Drug Metab. Dispos..

[46] Kim D.H., Ahn T., Jung H.C., Pan J.G., Yun C.H. (2009). Generation of the human metabolite piceatannol from the anticancer-preventive agent resveratrol by bacterial cytochrome P450 BM3. Drug Metab. Dispos..

[47] Jang H.H., Ryu S.H., Le T.K., Doan T.T.M., Nguyen T.H.H., Park K.D., Yim D.E., Kim D.H., Kang C.K., Ahn T. (2017). Regioselective C-H hydroxylation of omeprazole Sulfide by *Bacillus megaterium* CYP102A1 to Produce a Human Metabolite. Biotechnol. Lett..

[48] Le T.K., Jang H.H., Nguyen H.T.H., Doan T.T.M., Lee G.Y., Park K.D., Ahn T., Joung Y.H., Kang H.S., Yun C.H. (2017). Highly Regioselective hydroxylation of polydatin, a resveratrol glucoside, for one-step synthesis of astringin, a piceatannol glucoside, by P450 BM3. Enzyme Microb. Technol..

[49] Nguyen N.A., Jang J., Le T.K., Nguyen T.H.H., Woo S.M., Yoo S.K., Lee Y.J., Park K.D., Yeom S.J., Kim G.J. (2020). Biocatalytic production of a potent inhibitor of adipocyte differentiation from phloretin using engineered CYP102A1. J. Agric. Food Chem..

[50] Nguyen N.A., Cao N.T., Nguyen T.H.H., Le T.K., Cha G.S., Choi S.K., Pan J.G., Yeom S.J., Kang H.S., Yun C.H. (2020). Regioselective hydroxylation of phloretin, a bioactive compound from apples, by human Cytochrome P450 Enzymes. Pharmaceuticals.

[51] Fredenhagen A., Schroer K., Schröder H., Hoepfner D., Ligibel M., Porchet Zemp L., Radoch C., Freund E., Meishammer A. (2019). Cladosporin derivatives obtained by biotransformation provide guidance for the focused derivatization of this antimalarial lead compound. ChemBioChem.

[52] Venkataraman H., de Beer S.B.A., van Bergen L.A.H., van Essen N., Geerke D.P., Vermeulen N.P.E., Commandeur J.N.M. (2012). A Single Active Site Mutation Inverts Stereoselectivity of 16-hydroxylation of testosterone catalyzed by engineered cytochrome P450BM3. ChemBioChem.

[53] Reinen J., Vredenburg G., Klaering K., Vermeulen N.P.E., Commandeur J.N.M., Honing M., Vos J.C. (2015). Selective whole-cell biosynthesis of the designer drug metabolites 15- or 16-betahydroxynorethisterone by engineered cytochrome P450 BM3 Mutants. J. Mol. Catal. B Enzym..

[54] Venkataraman H., te Poele E.M., Rosłoniec K.Z., Vermeulen N., Commandeur J.N.M., van der Geize R., Dijkhuizen L. (2015). Biosynthesis of a steroid metabolite by an engineered *Rhodococcus Erythropolis* strain expressing a mutant cytochrome P450 BM3 enzyme. Appl. Microbiol. Biotechnol..

[55] Rea V., Kolkman A.J., Vottero E., Stronks E.J., Ampt K.A.M., Honing M., Vermeulen N.P.E., Wijmenga S.S., Commandeur J.N.M. (2012). Active site substitution A82W improves the regioselectivity of steroid hydroxylation by cytochrome P450 BM3 mutants as rationalized by spin relaxation nuclear magnetic resonance studies. Biochemistry.

[56] Cha G.S., Ryu S.H., Ahn T., Yun C.H. (2014). Regioselective hydroxylation of 17β-estradiol by mutants of CYP102A1 from *Bacillus megaterium*. Biotechnol. Lett..

[57] Liu X., Kong J.-Q. (2017). Steroids hydroxylation catalyzed by the monooxygenase mutant 139-3 from *Bacillus megaterium* BM3. Acta Pharm. Sin. B.

[58] Marchetti S., Schellens J.H.M. (2007). The impact of FDA and EMEA guidelines on drug development in relation to phase 0 trials. Br. J. Cancer.

[59] Food and Drug Administration Center for Drug Evaluation and Research (2016). Safety Testing of Drug Metabolites: Guidance for Industry.

[60] Kim D.H., Kim K.H., Kim D., Jung H.C., Pan J.G., Chi Y.T., Ahn T., Yun C.H. (2010). Oxidation of human cytochrome P450 1A2 substrates by *Bacillus megaterium* cytochrome P450 BM3. J. Mol. Catal. B Enzym..

[61] Rentmeister A., Brown T.R., Snow C.D., Carbone M.N., Arnold F.H. (2011). Engineered Bacterial Mimics of Human Drug Metabolizing Enzyme CYP2C9. ChemCatChem.

[62] Tsotsou G.E., Sideri A., Goyal A., Di Nardo G., Gilardi G. (2012). Identification of mutant Asp251Gly/Gln307His of cytochrome P 450 BM3 for the generation of metabolites of diclofenac, ibuprofen and tolbutamide. Chem. A Eur. J..

[63] Di Nardo G., Dell’Angelo V., Catucci G., Sadeghi S.J., Gilardi G. (2016). Subtle structural changes in the Asp251Gly/Gln307His P450 BM3 mutant responsible for new activity toward diclofenac, tolbutamide and ibuprofen. Arch. Biochem. Biophys..

[64] Ren X., Yorke J.A., Taylor E., Zhang T., Zhou W., Wong L.L. (2015). Drug oxidation by cytochrome P450BM3: Metabolite synthesis and discovering new P450 reaction types. Chem. A Eur. J..

[65] Venkataraman H., Verkade-Vreeker M.C.A., Capoferri L., Geerke D.P., Vermeulen N.P.E., Commandeur J.N.M. (2014). Application of engineered cytochrome P450 mutants as biocatalysts for the synthesis of benzylic and aromatic metabolites of fenamic acid NSAIDs. Bioorg. Med. Chem..

[66] Butler C.F., Peet C., McLean K.J., Baynham M.T., Blankley R.T., Fisher K., Rigby S.E.J., Leys D., Voice M.W., Munro A.W. (2014). Human P450-like oxidation of diverse proton pump inhibitor drugs by “gatekeeper” mutants of flavocytochrome P450 BM3. Biochem. J..

[67] Le T.K., Cha G.S., Jang H.H., Nguyen T.H.H., Doan T.T.M., Lee Y.J., Park K.D., Shin Y., Kim D.H., Yun C.H. (2019). Regioselective hydroxylation pathway of tenatoprazole to produce human metabolites by *Bacillus megaterium* CYP102A1. Process Biochem..

[68] Richards L., Lutz A., Chalmers D.K., Jarrold A., Bowser T., Stevens G.W., Gras S.L. (2019). Production of metabolites of the anti-cancer drug noscapine Using a P450BM3 mutant library. Biotechnol. Rep..

[69] Koyani R.D., Vazquez-Duhalt R. (2018). Enzymatic activation of the emerging drug resveratrol. Appl. Biochem. Biotechnol..

[70] Luirink R.A., Dekker S.J., Capoferri L., Janssen L.F.H., Kuiper C.L., Ari M.E., Vermeulen N.P.E., Vos J.C., Commandeur J.N.M., Geerke D.P. (2018). A combined computational and experimental study on selective flucloxacillin hydroxylation by cytochrome P450 BM3 variants. J. Inorg. Biochem..

[71] Kim D.H., Kim K.H., Kim D.H., Liu K.H., Jung H.C., Pan J.G., Yun C.H. (2008). Generation of human metabolites of 7-ethoxycoumarin by bacterial cytochrome P450 BM3. Drug Metab. Dispos..

[72] Otey C.R., Bandara G., Lalonde J., Takahashi K., Arnold F.H. (2006). Preparation of human metabolites of propranolol using laboratory-evolved bacterial cytochromes P450. Biotechnol. Bioeng..

[73] Nguyen T., Yeom S.-J., Yun C.-H. (2021). Production of a human metabolite of atorvastatin by bacterial CYP102A1 peroxygenase. Appl. Sci..

[74] Fairhead M., Giannini S., Gillam E.M.J., Gilardi G. (2005). Functional characterisation of an engineered multidomain human P450 2E1 by molecular lego. J. Biol. Inorg. Chem..

[75] Dodhia V.R., Fantuzzi A., Gilardi G. (2006). Engineering Human Cytochrome P450 enzymes into catalytically self-sufficient chimeras using molecular lego. J. Biol. Inorg. Chem..

[76] Sadeghi S.J., Di Nardo G., Gilardi G. (2020). Chimeric cytochrome P450 3A4 used for in Vitro prediction of food–drug interactions. Biotechnol. Appl. Biochem..

[77] Castrignanò S., D’Avino S., Di Nardo G., Catucci G., Sadeghi S.J., Gilardi G. (2018). Modulation of the interaction between human P450 3A4 and B. *megaterium* reductase via engineered loops. Biochim. Biophys. Acta Proteins Proteom..

[78] Degregorio D., D’Avino S., Castrignanò S., di Nardo G., Sadeghi S.J., Catucci G., Gilardi G. (2017). Human cytochrome P450 3A4 as a biocatalyst: Effects of the engineered linker in modulation of coupling efficiency in 3A4-BMR chimeras. Front. Pharmacol..

[79] Zuo R., Zhang Y., Jiang C., Hackett J.C., Loria R., Bruner S.D., Ding Y. (2017). Engineered P450 biocatalysts show improved activity and regio-promiscuity in aromatic nitration. Sci. Rep..

[80] Mukherjee G., Nandekar P.P., Wade R.C. (2020). Electron transfer from cytochrome P450 reductase to cytochrome p450: Towards a structural and dynamic understanding. bioRxiv.

[81] Lechner A., Brunk E., Keasling J.D. (2016). The need for integrated approaches in metabolic engineering. Cold Spring Harb. Perspect. Biol..

[82] Urlacher V.B., Girhard M. (2019). Cytochrome P450 monooxygenases in biotechnology and synthetic biology. Trends Biotechnol..

[83] Dezvarei S., Lee J.H.Z., Bell S.G. (2018). Stereoselective hydroxylation of isophorone by variants of the cytochromes P450 CYP102A1 and CYP101A1. Enzyme Microb. Technol..

[84] Kaluzna I., Schmitges T., Straatman H., Van Tegelen D., Mü M., Schü M., Mink D. (2016). Enabling selective and sustainable p450 oxygenation technology. Production of 4-hydroxy-α-isophorone on kilogram scale. Org. Process Res. Dev..

[85] Ilie A., Harms K., Reetz M.T. (2018). P450-catalyzed regio- and stereoselective oxidative hydroxylation of 6-Iodotetralone: Preparative-scale synthesis of a key intermediate for Pd-catalyzed transformations. J. Org. Chem..

[86] Klaus T., Seifert A., Häbe T., Nestl B.M., Hauer B. (2019). An Enzyme cascade synthesis of vanillin. Catalysts.

[87] Dennig A., Weingartner A.M., Kardashliev T., Müller C.A., Tassano E., Schürmann M., Ruff A.J., Schwaneberg U. (2017). An enzymatic route to α-tocopherol synthons: Aromatic hydroxylation of pseudocumene and mesitylene with P450 BM3. Chem. A Eur. J..

[88] Weingartner A.M., Sauer D.F., Dhoke G.V., Davari M.D., Ruff A.J., Schwaneberg U. (2018). A hydroquinone-specific screening system for directed P450 evolution. Appl. Microbiol. Biotechnol..

[89] Munday S.D., Dezvarei S., Lau I.C.K., Bell S.G. (2017). Examination of Selectivity in the Oxidation of Ortho- and Meta-Disubstituted Benzenes by CYP102A1 (P450 Bm3) Variants. ChemCatChem.

[90] Carmichael A.B., Wong L.L. (2001). Protein engineering of *Bacillus megaterium* CYP102A1. The oxidation of polycyclic aromatic hydrocarbons. Eur. J. Biochem..

[91] Whitehouse C.J.C., Bell S.G., Tufton H.G., Kenny R.J.P., Ogilvie L.C.I., Wong L.L. (2008). Evolved CYP102A1 (P450BM3) variants oxidise a range of non-natural substrates and offer new selectivity options. Chem. Commun..

[92] Holec C., Hartrampf U., Neufeld K., Pietruszka J. (2017). P450 BM3-catalyzed regio- and stereoselective hydroxylation aiming at the synthesis of phthalides and isocoumarins. ChemBioChem.

[93] Wang Z.J., Renata H., Peck N.E., Farwell C.C., Coelho P.S., Arnold F.H. (2014). Improved Cyclopropanation activity of histidine-ligated Cytochrome P450 enables the enantioselective formal synthesis of levomilnacipran. Angew. Chemie Int. Ed..

[94] Li Y., Wong L.L. (2019). Multi-functional oxidase activity of CYP102A1 (P450BM3) in the oxidation of quinolines and tetrahydroquinolines. Angew. Chemie Int. Ed..

[95] Li Y., Qin B., Li X., Tang J., Chen Y., Zhou L., You S. (2018). Selective oxidations of cyperenoic acid by slightly reshaping the binding pocket of cytochrome P450 BM3. ChemCatChem.

[96] Acevedo-Rocha C.G., Gamble C.G., Lonsdale R., Li A., Nett N., Hoebenreich S., Lingnau J.B., Wirtz C., Fares C., Hinrichs H. (2018). P450-catalyzed regio-and diastereoselective steroid hydroxylation: Efficient directed evolution enabled by mutability Landscaping. ACS Catal..

[97] Chu L.L., Pandey R.P., Lim H.N., Jung H.J., Thuan N.H., Kim T.S., Sohng J.K. (2017). Synthesis of umbelliferone derivatives in *Escherichia Coli* and their biological activities. J. Biol. Eng..

[98] Chu L.L., Pandey R.P., Jung N., Jung H.J., Kim E.H., Sohng J.K. (2016). Hydroxylation of Diverse Flavonoids by CYP450 BM3 variants: Biosynthesis of eriodictyol from naringenin in whole cells and its biological activities. Microb. Cell Fact..

[99] Hong L.L., Kong J.Q. (2020). Altering the Regioselectivity of Cytochrome P450 BM3 variant M13 toward genistein through protein engineering and variation of reaction conditions. ACS Omega.

[100] Bisterfeld C., Holec C., Böse D., Marx P., Pietruszka J. (2017). Chemoenzymatic total synthesis of the proposed structures of putaminoxins B and D. J. Nat. Prod..

[101] Loskot S.A., Romney D.K., Arnold F.H., Stoltz B.M. (2017). Enantioselective total synthesis of Nigelladine A via Late-Stage C-H Oxidation Enabled by an Engineered P450 Enzyme. J. Am. Chem. Soc..

[102] Falck J.R., Reddy Y.K., Haines D.C., Reddy K.M., Krishna U.M., Graham S., Murry B., Peterson J.A. (2001). Practical, Enantiospecific Syntheses of 14,15-EET and Leukotoxin B (Vernolic Acid). Tetrahedron Lett..

[103] Zhang K., El Damaty S., Fasan R. (2011). P450 fingerprinting method for rapid discovery of terpene hydroxylating P450 catalysts with diversified regioselectivity. J. Am. Chem. Soc..

[104] Li J., Li F., King-Smith E., Renata H. (2020). Merging chemoenzymatic and radical-based retrosynthetic logic for rapid and modular synthesis of oxidized meroterpenoids. Nat. Chem..

[105] Dennig A., Marienhagen J., Ruff A.J., Guddat L., Schwaneberg U. (2012). Directed evolution of P450 BM3 into a P-xylene hydroxylase. ChemCatChem.

[106] Dennig A., Busto E., Kroutil W., Faber K. (2015). Biocatalytic one-pot synthesis of l-tyrosine derivatives from monosubstituted benzenes, pyruvate, and Ammonia. ACS Catal..

[107] Le-Huu P., Petrović D., Strodel B., Urlacher V.B. (2016). One-pot, two-step hydroxylation of the macrocyclic diterpenoid β-cembrenediol catalyzed by P450 BM3 Mutants. ChemCatChem.

[108] Wu S., Snajdrova R., Moore J.C., Baldenius K., Bornscheuer U.T. (2021). Biocatalysis: Enzymatic synthesis for industrial applications. Angew. Chemie Int. Ed..

[109] Chen W., Fisher M.J., Leung A., Cao Y., Wong L.L. (2020). Oxidative diversification of steroids by nature-inspired scanning glycine Mutagenesis of P450BM3 (CYP102A1). ACS Catal..

[110] Li A., Acevedo-Rocha C.G., D’Amore L., Chen J., Peng Y., Garcia-Borràs M., Gao C., Zhu J., Rickerby H., Osuna S. (2020). Regio- and stereoselective steroid hydroxylation at C7 by cytochrome P450 monooxygenase mutants. Angew. Chemie Int. Ed..

[111] Kolev J.N., O’Dwyer K.M., Jordan C.T., Fasan R. (2014). Discovery of potent parthenolide-based antileukemic agents enabled by late-stage P450-mediated C—H functionalization. ACS Chem. Biol..

[112] Alwaseem H., Frisch B.J., Fasan R. (2018). Anticancer activity Profiling of Parthenolide Analogs Generated via P450-mediated chemoenzymatic synthesis. Bioorg. Med. Chem..

[113] Zhang X., King-Smith E., Dong L.B., Yang L.C., Rudolf J.D., Shen B., Renata H. (2020). Divergent synthesis of complex diterpenes through a hybrid oxidative approach. Science.

[114] Kitamura E., Otomatsu T., Maeda C., Aoki Y., Ota C., Misawa N., Shindo K. (2013). Production of hydroxlated flavonoids with cytochrome P450 BM3 variant F87V and their antioxidative activities. Biosci. Biotechnol. Biochem..

[115] Ko S., Yang Y.H., Choi K.Y., Kim B.G. (2015). Rational design and directed evolution of CYP102A1 (BM3) for regio-specific hydroxylation of isoflavone. Biotechnol. Bioprocess Eng..

[116] Wang J.B., Ilie A., Reetz M.T. (2017). Chemo- and stereoselective cytochrome P450-BM3-catalyzed sulfoxidation of 1-Thiochroman-4-Ones enabled by directed evolution. Adv. Synth. Catal..

[117] Syntrivanis L.-D., Wong L.L., Robertson J. (2018). Hydroxylation of eleuthoside synthetic Intermediates by P450 _BM3_ (CYP102A1). Eur. J. Org. Chem..

[118] Vickers C., Backfisch G., Oellien F., Piel I., Lange U.E.W. (2018). Enzymatic Late-stage oxidation of lead compounds with solubilizing biomimetic docking/protecting groups. Chem. A Eur. J..

[119] Shoji O., Yanagisawa S., Stanfield J.K., Suzuki K., Cong Z., Sugimoto H., Shiro Y., Watanabe Y. (2017). Direct hydroxylation of benzene to phenol by cytochrome P450BM3 triggered by amino acid derivatives. Angew. Chemie Int. Ed..

[120] Karasawa M., Stanfield J.K., Yanagisawa S., Shoji O., Watanabe Y. (2018). Whole-cell biotransformation of benzene to phenol catalysed by intracellular Cytochrome P450BM3 activated by external additives. Angew. Chemie Int. Ed..

[121] Shoji O., Watanabe Y. (2017). Monooxygenation of nonnative substrates catalyzed by bacterial cytochrome P450s Facilitated by decoy molecules. Chem. Lett..

[122] Dubey K.K., Haque S., Jawed A., Singh B.P., Behera B.K. (2010). Construction of recombinant *Escherichia coli* for enhanced bioconversion of colchicine into 3-demethylated colchicine at 70l Bioreactor Level. Process Biochem..

[123] Yari M., Ghoshoon M.B., Vakili B., Ghasemi Y. (2017). Therapeutic enzymes: Applications and Approaches to Pharmacological Improvement. Curr. Pharm. Biotechnol..

[124] Shapiro M.G., Westmeyer G.G., Romero P.A., Szablowski J.O., Küster B., Shah A., Otey C.R., Langer R., Arnold F.H., Jasanoff A. (2010). Directed evolution of a magnetic resonance imaging contrast agent for noninvasive imaging of Dopamine. Nat. Biotechnol..

[125] Brustad E.M., Lelyveld V.S., Snow C.D., Crook N., Jung S.T., Martinez F.M., Scholl T.J., Jasanoff A., Arnold F.H. (2012). Structure-Guided Directed evolution of highly selective P450-based magnetic resonance imaging sensors for dopamine and serotonin. J. Mol. Biol..

[126] Vredenburg G., den Braver-Sewradj S., van Vugt-Lussenburg B.M.A., Vermeulen N.P.E., Commandeur J.N.M., Vos J.C. (2015). Activation of the anticancer drugs cyclophosphamide and ifosfamide by Cytochrome P450 BM3 Mutants. Toxicol. Lett..

[127] González-Davis O., Chauhan K., Zapian-Merino S.J., Vazquez-Duhalt R. (2020). Bi-enzymatic virus-like bionanoreactors for the transformation of endocrine disruptor compounds. Int. J. Biol. Macromol..

[128] Cirino P.C., Arnold F.H. (2003). A self-sufficient peroxide-driven hydroxylation biocatalyst. Angew. Chemie Int. Ed..

[129] Chauhan K., Sengar P., Juarez-Moreno K., Hirata G.A., Vazquez-Duhalt R. (2020). Camouflaged, activatable and therapeutic tandem bionanoreactors for breast cancer theranosis. J. Colloid Interface Sci..

